# Estimating the valence and arousal of dyadic conversations using autonomic nervous system responses and regression algorithms

**DOI:** 10.3389/fnrgo.2025.1671311

**Published:** 2025-12-03

**Authors:** Iman Chatterjee, Maja Goršič, Robert A. Kaya, Joshua D. Clapp, Vesna D. Novak

**Affiliations:** 1 Department of Electrical and Computer Engineering, University of Cincinnati, Cincinnati, OH, United States; 2 Department of Biomedical Engineering, Marquette University, Milwaukee, WI, United States; 3 Department of Psychology, University of Wyoming, Laramie, WY, United States

**Keywords:** affective computing, autonomic nervous system responses, conversation, dyads, physiological computing, psychophysiology, regression

## Abstract

**Introduction:**

Autonomic nervous system responses provide valuable information about interactions between pairs or groups of people but have primarily been studied using group-level statistical analysis, with a few studies attempting single-trial classification. As an alternative to classification, our study uses regression algorithms to estimate the valence and arousal of specific conversation intervals from dyads' autonomic nervous system responses.

**Methods:**

Forty-one dyads took part in 20-minute conversations following several different prompts. The conversations were divided into ten 2-minute intervals, with participants self-reporting perceived conversation valence and arousal after each 2-minute interval. Observers watched videos of the conversations and separately also rated valence and arousal. Four autonomic nervous system responses (electrocardiogram, electrodermal activity, respiration, skin temperature) were recorded, and both individual and synchrony features were extracted for each 2-minute interval. These extracted features were used with feature selection and a multilinear perceptron to estimate self-reported and observer-reported valence and arousal of each interval in both a dyad-specific (based on data from same dyad) and dyad-nonspecific (based on data from other dyads) manner.

**Results:**

Both dyad-specific and dyad-nonspecific regression using the multilinear perceptron resulted in lower root-mean-square errors than a simple median-based estimator and two other regression methods (linear regression and support vector machines).

**Discussion:**

The results suggest that physiological measurements can be used to characterize dyadic conversations on the level of individual dyads and conversation intervals. In the long term, such regression algorithms could potentially be used in applications such as education and mental health counseling.

## 1 Introduction

In the fields of affective computing and psychophysiology, physiological responses can be used to provide insight into diverse psychological processes, from cognitive workload (Vogl et al., 2025) and anger (Maillant et al., 2025) to enjoyment of games (Chanel et al., 2011) and exercises (Novak et al., 2011). Though most work in psychophysiology and affective computing has involved scenarios with a single participant, the last few years have seen more and more studies with dyads and groups involved in competition, collaboration, and conversation (Levenson, 2024). In such scenarios, it is possible to not only collect physiological data from each individual, but to also calculate the degree of physiological synchrony (also called synchronization, linkage, or hyperscanning in the case of central nervous system responses) between participants (Delaherche et al., 2012; Helm et al., 2018; Levenson, 2024; Schneider et al., 2020), potentially obtaining additional insight into interpersonal dynamics.

Dyadic physiological responses and physiological synchrony are known to be correlated with perceived cooperation quality (Balconi and Vanutelli, 2017; Hu et al., 2018), shared high-attention moments (Muszynski et al., 2018; Schmälzle and Grall, 2020), moments of interpersonal closeness during meditation (Ludwig et al., 2025), perceived therapist empathy and alliance in therapist-client interactions (Kleinbub et al., 2019; Tschacher and Meier, 2020), and engagement between teachers and students (Dikker et al., 2017; Sun et al., 2020; Zheng et al., 2020). Furthermore, synchrony patterns can differentiate leaders vs. followers (Jiang et al., 2015; Konvalinka et al., 2014), speakers vs. listeners (Dai et al., 2018) and neurotypical vs. autistic individuals (Dunsmore et al., 2019). However, most of this dyadic work has involved group-level statistical analysis: for example, showing significant physiological differences between leaders and followers or significant correlations between physiological and psychological variables. Only recently has there been a shift toward single-trial analysis: combining physiological responses with machine learning to identify the state of a single, specific interacting dyad. Such a shift from group-level statistical analysis to machine-learning-based recognition of specific individuals' states previously occurred in single-user affective computing (Picard et al., 2001), paving the way for extensive work in the field.

A few examples of single-trial dyad state recognition from physiological responses do exist and can be roughly divided into classification (assigning a discrete psychological class to a physiological measurement from a list of multiple possible classes) and regression (assigning a continuous value of a psychological variable to a physiological measurement). Both approaches commonly involve supervised machine learning: they learn the relationship between physiological and psychological variables based on previously recorded and labeled training data. The same division has historically existed in single-user affective computing, where classification into 2–4 classes (e.g., low/medium/high stress) has been much more popular than regression (e.g., assigning a stress value from 0 to 100) (Novak et al., 2012)—perhaps because classification study designs are simpler and classification results (presented as accuracy in percentages) are much easier to interpret.

Among dyadic classification studies, most studies have used supervised machine learning with a single physiological modality (e.g., only heart rate) to discriminate between two (Brouwer et al., 2019; Hernandez et al., 2014; Jamil and Belkacem, 2024; Konvalinka et al., 2014; Muszynski et al., 2018; Pan et al., 2020; Sharika et al., 2024; Zhang et al., 2024), three (Simar et al., 2020; Zhang et al., 2024) or four classes (Verdiere et al., 2019). Relatively few studies have performed classification using multiple physiological modalities; among them, notably, our own team conducted two studies (one with a competition scenario, one with conversation) and found that classification accuracy is higher with multiple modalities than a single modality (Chatterjee et al., 2023; Darzi and Novak, 2021). Instead of supervised classification, one study used unsupervised clustering into two classes (Stuldreher et al., 2022) and also found higher accuracy when multiple modalities were used.

Dyadic regression studies are less common than dyadic classification studies, and we are aware of only two examples. One study used electroencephalography to estimate arousal and valence on 1–9 scales while participants watched videos together (Ding et al., 2021). Our own previous study tried to estimate engagement in a freeflowing conversation on a 0–100 scale (Chatterjee et al., 2021), though aspects of the design likely impacted the quality of results. For example, as little guidance was given regarding conversation topics, participants tended to exhibit a limited range of engagement. Additionally, the sample in that initial study (16 dyads) was small, questioning the degree to which regression algorithms could receive adequate training. Finally, the 0–100 scale was *ad-hoc* and had not been validated, questioning the degree to which scores reliably captured engagement. The limitations of this prior regression work (Chatterjee et al., 2021) therefore informed our current study.

While we believe that classification is likely to remain more popular in dyadic affective computing, regression nonetheless has some potential advantages—for example, the ability to provide more granular feedback to users. The current study is thus inspired by our previous regression work (Chatterjee et al., 2021) but uses a redesigned study protocol as well as a much larger sample size. Its primary goal is to determine how accurately the valence and arousal of dyadic conversation intervals can be estimated from autonomic nervous system responses in three ways:

- Estimating the conversation valence/arousal perceived by each participant, using regression algorithms trained on labeled physiological data from the same dyad,
- Estimating the conversation valence/arousal perceived by each participant, using regression algorithms trained on labeled physiological data from other dyads,
- Estimating the conversation valence/arousal perceived by external observers, using regression algorithms trained on labeled physiological data from other dyads.

**Figure 1:**
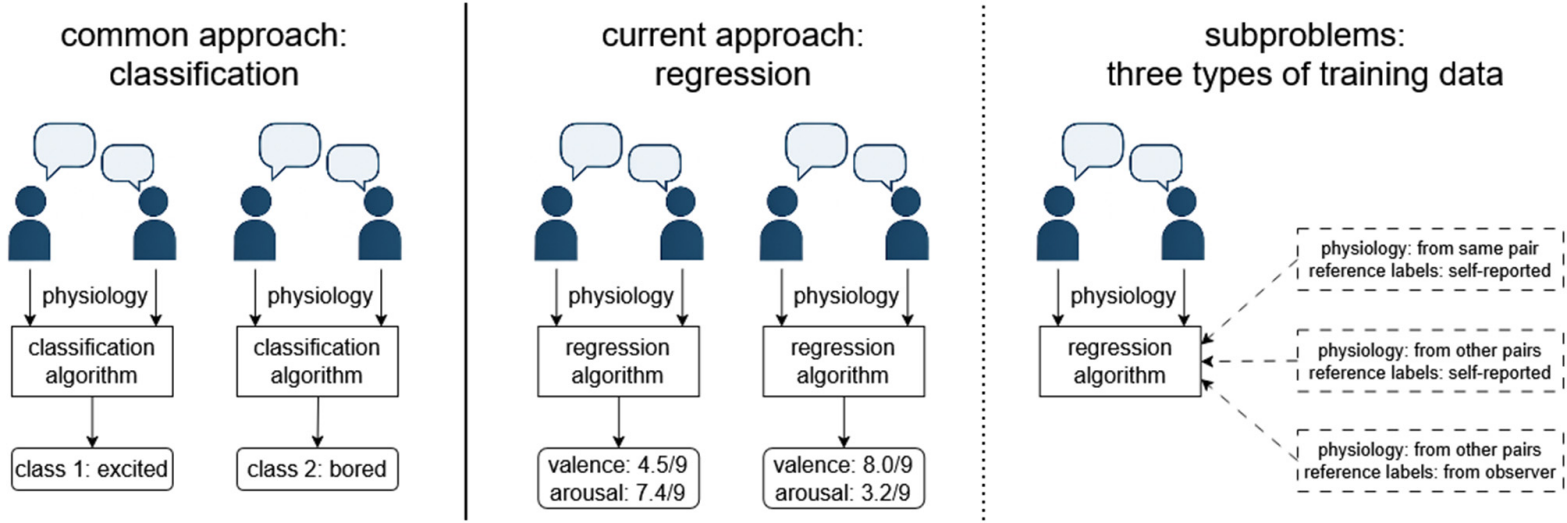
The overall study concept. While several studies have been conducted on classification of dyadic physiological responses, our study focuses on regression. In the primary analysis, there are three subproblems depending on whether training data are obtained from the same dyad or from other dyads and whether reference labels are provided by the participants or by observers.

The overall concept of the work and these three subgoals are illustrated in Figure 1. The reasoning for these three subgoals was as follows: In single-user affective computing, supervised machine learning is much more common than unsupervised methods (Aranha et al., 2021; Novak et al., 2012), leading to our focus on regression algorithms trained on labeled data given the limited state of the art in dyadic regression. Despite numerous weaknesses, the use of self-report data for training is also the dominant paradigm in single-user affective computing (Aranha et al., 2021; Novak et al., 2012), with the idea being that the algorithm is trained based on data from an initial “calibration” period with the same dyad (Goal 1) or based on a previously collected dataset of other dyads (Goal 2), then infers the conversation state on its own using only unlabeled physiological data. However, given the known weaknesses of self-report data (e.g., having to interrupt participants, participants being unwilling to accurately report negative states), observer ratings represent a somewhat popular alternative to self-report and were highlighted as a possible alternative in our previous study (Chatterjee et al., 2021); they were thus investigated for Goal 3.

Positive results of the study (accurate estimation of conversation valence/arousal) would indicate that such regression algorithms could potentially be used to extract dyadic and group states from autonomic nervous system responses in diverse applications. They could provide real-time feedback during conversations—for example, by visualizing the client's degree of trust to novice therapists (Bar-Kalifa et al., 2019; Kleinbub et al., 2019; Tschacher and Meier, 2020) or by visualizing students' engagement levels to novice teachers (Dikker et al., 2017; Sun et al., 2020; Zheng et al., 2020). They could also be used to analyze conversations post hoc—for example, by evaluating teachers' abilities to engage students after the lecture has concluded. Furthermore, the algorithms could be modified for cooperation or competition scenarios.

Early results of the study were published as a preliminary conference paper (Chatterjee et al., 2024), which involved only partial results of the third approach (valence/arousal perceived by external observers) on part of the full dataset with less effective algorithms.

## 2 Materials and methods

The study was approved by the Institutional Review Board of the University of Cincinnati, protocol 2021-1107. Participating dyads (Section 2.1) took part in a single session. The study protocol included an initial 2-min baseline interval in which participants rested quietly, ten 2-min conversation intervals where dyads talked to each other according to different prompts, and a final 2-min baseline interval that involved a second period of quiet rest (Section 2.2). After each 2-min conversation interval, participants recorded their perception of the interaction using the Self-Assessment Manikin (SAM) (Section 2.3). External observers then watched videos of the conversations and also rated their perception of them using the SAM (Section 2.4). Physiological data were collected throughout these intervals (Section 2.5). The overall goal of the study was to use machine learning to estimate self-reported and observer-reported SAM ratings (Section 2.6) based on physiological data. Additionally, three secondary analyses were conducted (Section 2.7).

### 2.1 Participants

Participants were recruited via e-mail advertisements sent to University of Cincinnati students and employees. Participants were encouraged to volunteer with a self-selected partner; any solo volunteers were paired with another solo volunteer.

Forty-two dyads took part in the study; one dyad's data were discarded due to signal issues, resulting in 41 valid dyads for analysis. Participants in 32 dyads were acquainted before participation. There were 16 male-female dyads, 12 female-female dyads, 10 male-male dyads, two female-nonbinary dyads, and one nonbinary-nonbinary dyad. Participants were 21.1 ± 3.8 years old (mean ± standard deviation), with the range being 18–33.

All participants provided self-report scores for four traits known to influence physiological synchrony: cognitive and affective empathy with the Questionnaire of Cognitive and Affective Empathy (Reniers et al., 2011), social anxiety with the Brief Fear of Negative Evaluation Scale (Leary, 1983), and depression with the Center for Epidemiologic Studies Depression Scale (Radloff, 1977). Their scores were: 55.7 ± 8.9 for cognitive empathy (possible range 19–95), 34.0 ± 5.4 for affective empathy (possible range 12–60), 39.8 ± 10.1 for social anxiety (possible range 12–60), and 17.2 ± 11.4 for depression (possible range 0–60). In all cases, higher scores indicate higher empathy/anxiety/depression. Copies of all questionnaires are available in the online data repository (see Data availability statement).

### 2.2 Study protocol

**Figure 2:**
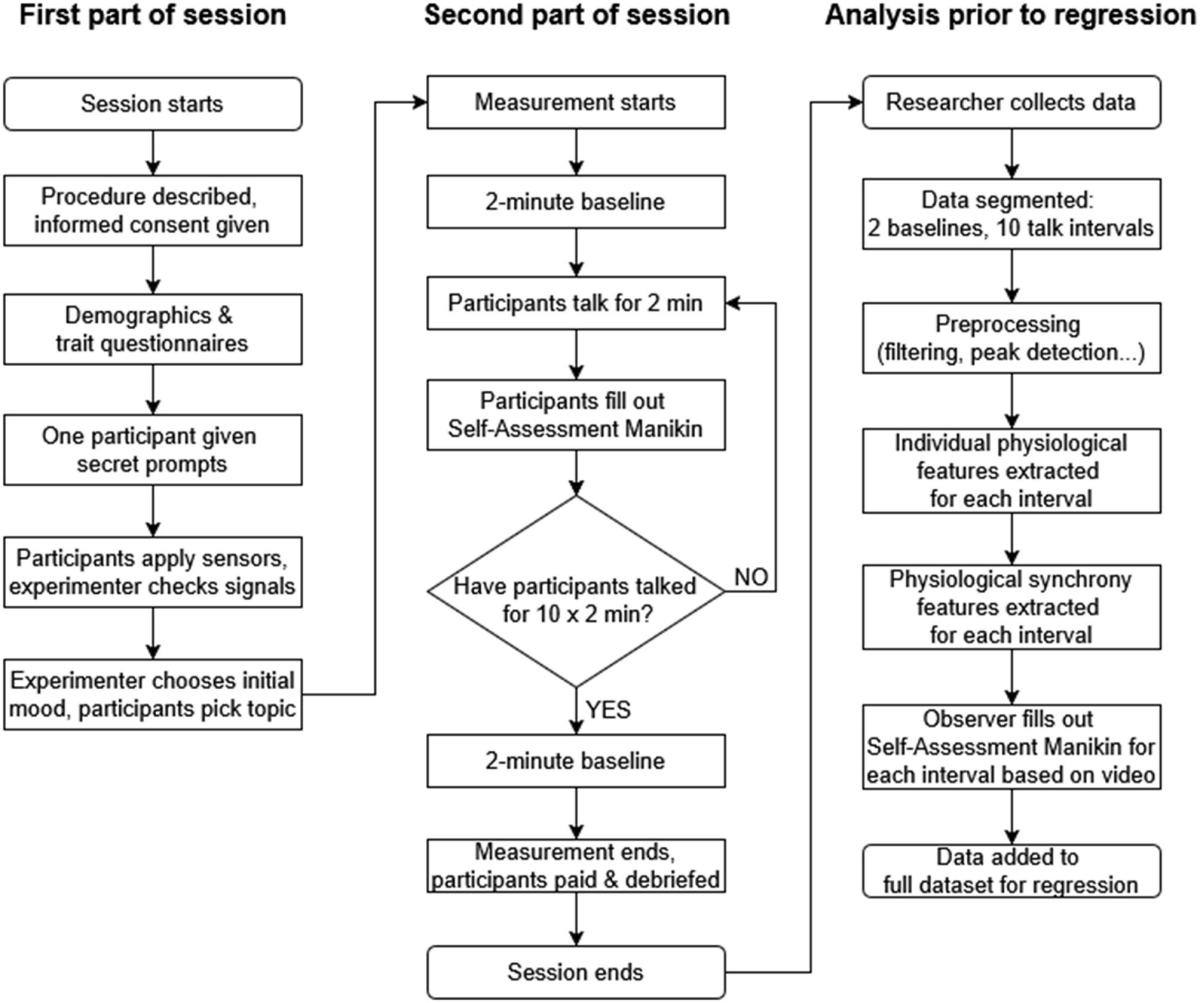
The study protocol and the data analysis steps prior to regression.

The protocol built on our previous study on estimation of interpersonal engagement from physiological responses (Chatterjee et al., 2021), but with several modifications. Each dyad took part in a 75-min session, with the individual session steps summarized in Figure 2. Upon arrival, the purpose and procedure of the experiment were explained. Participants signed an informed consent form, provided basic demographic information, and filled out trait questionnaires (Section 2.1). After completing these questionnaires, one member of the dyad was briefly taken to a side room with a request to “clarify questionnaire answers”; during this time, researchers informed this participant about secret prompts to encourage solicitation of more negative interactions during predetermined intervals (see below).

**Figure 3:**
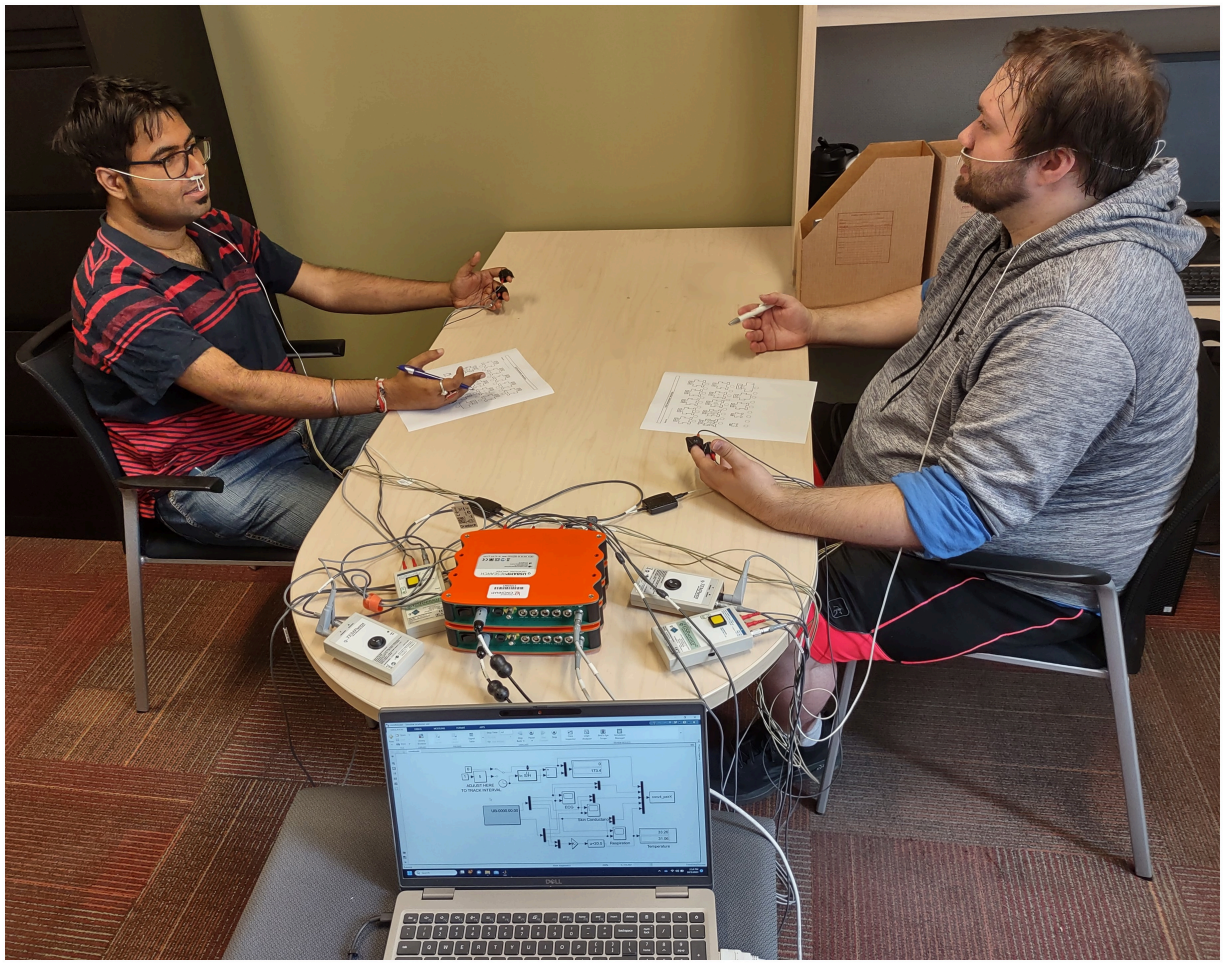
Photo of the experiment session. Two participants sit facing each other wearing physiological sensors, with questionnaires placed on the table between them. The experimenter sits to the side with the data collection laptop; the photo is taken from their perspective. The signal amplifiers are on the table between the laptop and participants, and webcams and a microphone on the table are used to collect video and audio. The photo was published as part of our preliminary work (Chatterjee et al., 2024) and is reused with permission.

For the experiment, participants sat facing each other on opposite sides of a table approximately 1.5 m apart, with the experimenter positioned to the side (Figure 3). Physiological sensors were self-applied under the direction of the experimenter, who visually checked signal quality and provided feedback until good signal quality was obtained. Such self-application was originally introduced in our previous studies due to COVID-19 (Chatterjee et al., 2021, 2023) and retained for consistency.

After sensor application, dyads were randomly assigned one of three initial conversation topics: positive, neutral, or negative. The positive group was asked to begin conversation by talking about a self-selected topic that both participants liked and agreed on (e.g., shared enthusiasm about hobbies or media). The negative group was asked to begin by talking about a self-selected topic that participants disagreed on (e.g., opinions on sports teams, or food). Specific topics were selected by participants and written down by the experimenter before proceeding; while no specific topics were forbidden, participants tended to self-select “trivial” topics and avoid issues such as politics. The neutral group began by talking about career goals. The topic of career goals was selected based on our previous study where all participants started with this topic (Chatterjee et al., 2021); positive and negative options were inspired by our previous study on acted conversation scenarios (Chatterjee et al., 2023) and other dyadic studies (Levenson, 2024).

Once the topic was selected, participants sat quietly with eyes open for the 2-min initial baseline, then engaged in ten 2-min conversation intervals. Dyads were instructed to begin the first interval by talking about the topic assigned to their group but were told that this was only an initial prompt and that they may switch to whatever topic they desire at any time after the first few sentences. At the end of each 2-min interval, the experimenter raised a hand to signal participants to quickly provide impressions of the previous 2-min conversation interval using paper SAM forms (Section 2.3). After this self-report, participants continued the conversation and the experimenter marked the start of the next conversation interval. After the tenth interval, participants sat with eyes open for another 2 minutes to obtain a second baseline measurement. They removed sensors, were thanked for their time, and received $15 Amazon gift cards as compensation.

To increase the range of psychological states in the study protocol, one member of the dyad was informed about two “secret prompts” at the start of the session (see above); the other participant was not aware of them until the session concluded. These prompts (“During the next 2-min interval, point out every possible flaw with whatever the other person says” and “During the next 2-min interval, show absolutely no emotion”) were presented after the fourth and seventh 2-min interval, with the order counterbalanced across dyads. Prompts were displayed on the paper SAM form provided to the selected dyad member in text that was large enough for the participant to notice but not so obvious that their partner would notice as well. Prompts were specifically intended to introduce more negative responses to the interaction since our previous regression study found mostly positive states during freeflowing conversations (Chatterjee et al., 2021). The “point out every flaw” prompt is an example of excessive disagreement/criticism while the “show no emotion” prompt is based on prior work that found negative reactions to emotion suppression in conversation partners (Butler et al., 2003).

### 2.3 Self-Assessment Manikin

Both participants completed the SAM for each 2-min conversation interval. The original SAM (Bradley and Lang, 1994) uses three graphical items to measure individual valence, arousal and dominance on 9-point scales; the dominance item is often omitted. In our study, the valence and arousal items were presented as in the original SAM, but participants were instructed to rate the valence and arousal of the overall conversation, not only their contribution to it. The dominance item was replaced by a new ‘balance' item, but this ‘balance' item was found to be difficult to interpret by participants and is thus not analyzed in the current paper, though raw data are available in the online data repository (see Data availability statement).

The SAM was selected since it is very common in affective computing and was used in a prior dyadic conversation study by other authors (Ding et al., 2021). Our previous conversation study, which inspired this protocol, used an entirely *ad-hoc* questionnaire (Chatterjee et al., 2021), and the results were difficult to interpret as a result, leading to the choice of a standard questionnaire. Performing the SAM generally took 10–20 s after the first interval and 5–10 s after other intervals when participants already had practice with it; participants were usually able to return to the previous conversation topic with little disruption.

### 2.4 Observer analysis of audio/video data

Throughout the conversation, audio and video of the participants were recorded with a Yeti X microphone (Blue Microphones, USA) and two webcams placed on the table between the participants, with one webcam pointed at each participant's face (Figure 3). Recordings from one of the 41 dyads were lost, resulting in 40 dyads' audio/video recordings for analysis.

The recordings were divided into 10 segments corresponding to the 10 2-min conversation intervals. Each 2-min segment was then reviewed by two independent researchers who were not involved in data collection or analysis of physiological data. These observers rated the valence and arousal of the overall conversation in each interval using the same SAM used by participants (Section 2.3). Behavioral anchors for observer ratings of valence and arousal at the dyad level were developed using video from 5 randomly selected dyads. Intervals from an additional three randomly selected dyads (30 unique intervals) were used to verify the consistency of ratings across coders before finalizing scores for the remaining dyads. Nine of the remaining cases were selected at random to be coded by both reviewers as a formal assessment of interrater reliability. Estimates of consistency were excellent for valence (ICC = 0.87) and arousal (ICC = 0.81) codes.

The 5 dyads used for development of behavioral anchors were not used for regression of observer-rated valence and arousal from physiological data. Thus, the final dataset used for regression of observer-rated valence and arousal (Section 2.6) consisted of data from 35 dyads.

### 2.5 Physiological sensors and feature extraction

The sensors were the same as those used in our previous study (Chatterjee et al., 2021) and consisted of four autonomic nervous system responses collected from both participants in the dyad. Since the focus of our research is on physiological responses, we did not include other modalities such as speech or facial expressions in the analysis, though this would be a possible future goal. We did test electroencephalography as an additional measurement in initial pilot sessions since it is popular in dyadic affective computing (Ding et al., 2021; Simar et al., 2020; Verdiere et al., 2019), but eventually chose not to include it due to the longer setup time and significant motion artifacts during conversation.

Two g.USBamp amplifiers (g.tec Medical Engineering GmbH, Austria) and associated g.tec sensors were used to record four signals from each participant at 600 Hz: the electrocardiogram (ECG—via four disposable electrodes on the torso), electrodermal activity (EDA—via two electrodes on the distal phalanges of the index and middle fingers of the nondominant hand), respiration (via a thermistor-based sensor under the nose), and skin temperature (via a dry electrode on the distal phalanx of the little finger of the nondominant hand). All signals were analog and digital filtered. For details on hardware and filtering, see our previous paper (Chatterjee et al., 2021).

Filtered signals were segmented into 2-min baseline and conversation intervals, with a total of 12 intervals per dyad (2 baselines + 10 conversation intervals). Data not corresponding to these 12 intervals (e.g., questionnaire breaks between intervals) were discarded. In each interval, we first detected peaks in ECG, EDA, and respiration signals using standard peak detection algorithms reused from our previous study (Chatterjee et al., 2023). Detected ECG peaks were manually inspected to verify that they truly represented R-waves in ECG. Then, several features were extracted from each 2-min interval. These consisted of individual physiological features and synchrony features.

Individual physiological features were calculated from one participant's signal, and each feature existed separately for each of the two participants in an interval. They consisted of:

- ECG: mean heart rate, minimum heart rate, maximum heart rate, standard deviation of interbeat intervals, root mean square value of consecutive differences between interbeat intervals, percentage of consecutive interbeat intervals with a difference greater than 50 ms, power in low-frequency band, power in high-frequency band, and the ratio of the two powers (LF/HF ratio). These features are standard measures of heart rate variability and can be used with intervals that are at least 2 minutes long (Task Force of the European Society of Cardiology the North American Society of Pacing Electrophysiology, 1996).
- Respiration: mean respiration rate and standard deviation of respiration rate.
- EDA: mean EDA, final EDA, difference between initial and final EDA values, number of skin conductance responses, mean skin conductance response amplitude, standard deviation of skin conductance response amplitudes. Skin conductance responses were detected using an algorithm from our previous work (Darzi and Novak, 2021) and were considered valid if they were at least 0.05 microsiemens higher than the previous valley and occurred within 5 s of that valley.
- Skin temperature: mean temperature, final temperature, and difference between initial and final values.

Synchrony features were calculated based on the same signal of both participants in the dyad (e.g., both respiration signals) and thus do not exist separately for individual participants. They were calculated from filtered EDA and skin temperature signals as well as from instantaneous heart rate and respiration rate signals [computed as a function of time from raw electrocardiogram and nose respiration signals (Darzi and Novak, 2021)]. These instantaneous heart/respiration rate signals were used instead of raw signals since they exhibit higher synchrony between participants (Darzi and Novak, 2021). The same synchrony features were calculated for each of the four signal modalities. They were:

- Dynamic time warping (DTW) distance, as introduced by Muszynski et al. (2018) and used in our previous study (Chatterjee et al., 2023). Code for calculation was reused from our previous study (Chatterjee et al., 2023), and the feature is explained in detail there. The code is also available open-source as a supplement to that study (Chatterjee et al., 2022).
- Nonlinear interdependence, as introduced by Muszynski et al. (2018) and used in our previous study (Chatterjee et al., 2022, 2023).
- Coherence, with the method previously used by Darzi and Novak (2021) and in our previous study (Chatterjee et al., 2022, 2023).
- Cross-correlation, with the method previously used by Darzi and Novak (2021) and in our previous study (Chatterjee et al., 2022, 2023).
- Derivative DTW distance, as introduced by Keogh and Pazzani (2000, 2001). Classic DTW is limited in the sense that it only considers y-axis values of the signal in the timeseries while ignoring whether the signal has a rising or falling trend; in contrast, derivative DTW operates on local derivatives of the data rather than raw data and is more robust to outliers.
- Complexity-invariant DTW distance, as introduced by Batista et al. (2011), which is a variant of DTW that is immune to domain-dependent issues. For example, a slight variation in offset or scale between two timeseries tends to dominate classic DTW. Complexity-invariant DTW corrects for such issues using a factor that corrects for differences in complexity between two timeseries.
- Cosine distance, with the method of Nakamura et al. (2013). This is a simple measure of the cosine of the angle between the two vectors represented by the two timeseries.
- Hausdorff distance, with the method of Ryu and Kamata (2021). Hausdorff distance measures the max-min distance between two sets of points and captures the extent of resemblance between the two sets without having to define the correlation between them. It has been found to be useful in evaluating high-resolution data and takes into account the spatial relationship between the compared point sets as well as their overlaps.
- Symmetric segment path distance, with the method of Salarpour and Khotanlou (2019). This method captures direction-based changes in the segmented sub-trajectories. It calculates the minimum point-to-segment distance for every point of the first trajectory in all segments of the other one and then reports the mean of the computed distance for every trajectory point as the overall feature.

This list represents more features than were used in most previous studies on classification or regression of dyadic physiological responses. However, we chose to include more in order to examine their usefulness (as described in the next sections) and thus guide future research.

### 2.6 Primary analysis: estimation of self- and observer-reported valence and arousal

Data collected from each dyad included participant characteristics (age, gender, four personality traits), extracted physiological features (from first baseline + 10 conversation intervals + second baseline), self-reported psychological variables (valence and arousal, from 10 conversation intervals), and observer-reported psychological variables (valence and arousal, from 10 conversation intervals). There were then three goals in the primary analysis:

- Goal 1: Estimate the conversation valence/arousal perceived by each participant, using individual physiological features of that participant as well as the dyad's synchrony features,
- Goal 2: Estimate the conversation valence/arousal perceived by each participant, using individual physiological features and synchrony features from participants in other dyads,
- Goal 3: Estimate the conversation valence/arousal perceived by external observers, using individual physiological features and synchrony features from participants in other dyads.

In all three cases, physiological data included both individual and synchrony features. Participant characteristics were not used in primary analyses. Goals 1 and 2 were achieved with data from all 41 dyads while goal 3 was achieved with data from 35 dyads (since one dyad's videos were lost and 5 dyads were used to develop behavioral anchors). Additionally, we did not estimate observer-reported valence/arousal using data from the same dyad since observers must necessarily use data from multiple dyads to perform ratings.

Since valence and arousal ratings were done on a numerical scale (1–9), relating physiology to psychology represents a multivariate regression problem that can be solved with many different algorithms. While our study initially tested several regression methods, we overall achieved the best results with a multilayer perceptron (MLP). We thus present results for three regression methods:

- MLP, a relatively simple feedforward neural network that was implemented using the *feedforwardnet* function in MATLAB 2024. The MLP had a learning rate of 0.1 and was trained for 1,000 epochs, with Levenberg-Marquardt optimization used to update weights. As mentioned, it is presented because it achieved the highest performance for all three goals.
- Multiple linear regression with least squares fitting, implemented using the *fitrlinear* function in MATLAB. and regularized using lasso or ridge methods (implemented using *lasso* and *ridge* functions in MATLAB). Regularization techniques use penalty terms in the model's cost function to help in better omission of irrelevant features (Friedrich et al., 2023). Linear regression is presented since it is perhaps the best-known regression approach.
- Support vector machine (SVM) with a Gaussian kernel, implemented using the *fitrsvm* function in MATLAB. SVM with a Gaussian kernel is presented as a classic nonlinear approach with a long history of use in affective computing (Novak et al., 2012).

All three regression methods were preceded by feature selection to reduce input dimensionality. Two feature selection methods were tested for this purpose: maximum relevance minimum redundancy (MRMR, a supervised method implemented using MATLAB's *fsrmrmr* function) and ranking using Laplacian scores (an unsupervised method implemented using MATLAB's *fsulaplacian* function).

The supervised regression algorithms require training data: physiological features (inputs) together with known valence/arousal value (outputs). In affective computing, it is common to assume that the values reported by participants or observers are “true” and can be used to train the algorithms (Aranha et al., 2021; Novak et al., 2012). However, the training data can be either from the same dyad (dyad-specific—as in Goal 1) or other dyads (dyad-nonspecific—as in Goals 2 and 3) (Chatterjee et al., 2021). Therefore, two algorithmic approaches were used: dyad-specific and dyad-nonspecific regression.

#### 2.6.1 Dyad-specific regression

Dyad-specific regression was used to achieve Goal 1 and evaluated whether the conversation valence/arousal reported by a participant can be estimated based on training data from the same dyad: the participant's individual physiological features and the synchrony features from that dyad. The other participant's individual features were not used to estimate the valence/arousal reported by the first participant (and vice versa).

The regression was done using crossvalidation and an 8:1:1 training/validation/test split: for each participant, data from 8 “training” conversation intervals of the same dyad were used to train the regression algorithms, and different iterations of algorithms as well as different feature selection methods were compared on a ninth “validation” interval to check accuracy and identify the most effective algorithm parameters. Once the algorithms were fully trained and the most effective algorithm had been identified using the validation interval, the finalized algorithm was applied to the tenth “test” interval to estimate valence/arousal. This procedure was repeated 10 times, with each interval serving as the “test” interval once. Once done for one participant in the dyad, the procedure was repeated for the other participant in the dyad, then repeated in the same way for all dyads. Data from one dyad did not influence regression for other dyads at all. In single-user affective computing, such user-specific regression is popular when there is significant intersubject variability and training data therefore do not generalize to other participants (Aranha et al., 2021; Novak et al., 2012). However, we expected a priori that such user-specific regression would result in significant overfitting due to the small training dataset size (8 data points) and large number of features.

In addition to the supervised machine learning methods (MLP, linear regression, SVM), a simpler method was also used for comparison. In the simpler method (hereafter dubbed the median-based estimator), the valence/arousal in an interval was estimated as the median of valence/arousal values reported by that participant in the other 9 intervals of that dyad.

#### 2.6.2 Dyad-nonspecific regression

Dyad-nonspecific regression was used to achieve Goals 2 and 3 and evaluated whether the conversation valence/arousal reported by a participant or observer can be estimated based on training data from other dyads. This was again done using crossvalidation, with the regression algorithms trained using data from 40 dyads (Goal 2) or 34 dyads (Goal 3).

Specifically, to achieve Goal 2 (estimate valence/arousal perceived by each participant), we used crossvalidation with a 39:1:1 training/validation/test split: data from 78 “training” participants (39 dyads x 2 participants) were used to train the algorithms, with each data point consisting of individual physiological features from a participant and the synchrony features of the corresponding dyad (inputs) as well as the conversation valence/arousal reported by that participant for that conversation interval (outputs). Different iterations of algorithms and different feature selection methods were compared on a 40th “validation” dyad to check accuracy and identify the most effective algorithm parameters. Once the regression algorithm was finalized, it was applied to the 41^st^ “test” dyad to estimate valence/arousal. This was done 41 times, with each dyad serving as the “test” dyad once. In single-user affective computing, user-nonspecific methods often yield lower accuracies than user-specific methods due to lack of data from the same user (Aranha et al., 2021; Novak et al., 2012). However, in our study, the dyad-nonspecific training dataset size (780 samples) was much bigger than the dyad-specific case (8 samples), so we expected that the difference may not be as pronounced.

To achieve Goal 3 (estimate valence/arousal perceived by an observer), a 33:1:1 training/validation/test split was done due to the smaller sample size. This time, each data point consisted of the individual physiological features of both participants in the dyad and the synchrony features of the corresponding dyad (inputs) as well as the conversation valence/arousal reported by the observer for that conversation interval (outputs).

As different dyads may have different physiological responses, each dyad's physiological features in the 10 conversation intervals were normalized by subtracting the corresponding feature values from the first baseline interval of that dyad. Baseline subtraction is a common normalization approach in affective computing (Aranha et al., 2021; Novak et al., 2012). For full disclosure, we also tested normalization by subtracting the average of both baseline intervals, but this did not affect results.

As in dyad-specific regression, a simpler median-based estimator was also used for comparison. In Goal 2, the valence/arousal reported by a participant for the 10 intervals of that dyad was estimated as the median of self-reported valence/arousal values from the 80 participants in the 40 other dyads. In Goal 3, the valence/arousal reported by an observer for the 10 intervals of that dyad was estimated as the median of observer-reported valence/arousal values in the other 34 dyads.

#### 2.6.3 Performance evaluation

Both dyad-specific and dyad-nonspecific regression output valence and arousal values for each conversation interval. As self- or observer-reported valence/arousal values are assumed to be correct and are used to train the regression algorithms, the performance of the trained algorithms can be evaluated as the ability to match self- or observer-reported valence/arousal values. In each conversation interval, an individual valence/arousal estimation error was therefore defined as the difference between the value output by a regression algorithm and the participant's or observer's reported value for that interval. Two outcome values were then defined for each participant (Goals 1 and 2) or dyad (Goal 3): mean absolute (MA) error and root-mean-square (RMS) error across the 10 intervals of that participant/dyad. The same MA and RMS metrics were used in our previous work (Chatterjee et al., 2021); MA and RMS errors have also been used to evaluate regression in single-user affective computing (Bailenson et al., 2008), and MA errors have been used in another dyadic regression study (Ding et al., 2021).

In Goals 1 and 2 (which involve self-reported arousal/valence values), MA and RMS errors were compared between the four regression methods (MLP, linear regression, SVM, median-based estimator) using one-way repeated-measures analyses of variance (RMANOVA) with four levels (the four regression methods) and 82 samples (41 dyads x 2 participants) followed by *post-hoc* Holm-Sidak tests. In Goal 3, the same approach was used, but there were only 35 samples for 35 dyads. A priori, our expectations were:

- At least one of the three machine-learning based methods should achieve a significantly lower error than the median-based estimator. Failure to meet this expectation would mean that the machine-learning-based methods can do no better than a simple guess that the conversation is “about average”.
- At least one of the three machine-learning-based methods should achieve a mean MA error below 1.0 and RMS error below 1.5. Errors in this range were noted in prior single-user regression work (Bailenson et al., 2008) and the only other dyadic regression study (Ding et al., 2021). While the current work might still achieve higher errors due to, e.g., a different study setting, we felt that this would be a reasonable initial target.

### 2.7 Secondary analyses

In the primary analyses, all physiological features (individual and synchrony) were used as inputs to regression algorithms. However, physiological synchrony features can be computationally complex compared to better-established individual features and should be included only if they positively impact regression performance. As the first secondary analysis, we thus performed the same dyad-specific and dyad-nonspecific regression using the MLP algorithm, but with synchrony features removed from the input dataset. Paired *t*-tests or Wilcoxon tests were used to compare MA and RMS errors of MLP with and without synchrony features. Only the MLP was used in this analysis since it (as described later) outperformed multiple linear regression and SVMs in the primary analyses.

Next, since physiological synchrony is influenced by personality traits (Sachs et al., 2020; Steiger et al., 2019), providing explicit information about these traits may improve estimation performance. However, as personality questionnaires can be long and time-consuming, they should again only be administered if they improve performance. In the second secondary analysis, we thus included participant characteristics (age, gender, personality traits) as additional inputs to dyad-nonspecific estimation. These characteristics were the same for all 10 conversation intervals of a dyad but differed between dyads. The analysis was not conducted with dyad-specific estimation, where algorithms are already tailored to individual dyads. Again, this secondary analysis only used the MLP rather than linear regression or SVMs.

Finally, the most important features for regression of self-reported and observer-reported valence and arousal were identified for dyad-nonspecific estimation. These were selected as the top 5 features selected for valence and arousal by Laplacian feature ranking on the full dataset (41 dyads for self-reported, 35 for observer-reported). This was not done for dyad-specific estimation, where many more regression models are created and there is much more variance in predictor importance. Laplacian feature ranking was used over MRMR since it consistently resulted in better performance in dyad-nonspecific estimation (see next section).

## 3 Results

Individual participants' demographics, extracted physiological features, and questionnaire answers are available in the online data repository (see Data availability statement).

As a basic characterization of the self-report data:

- Means ± standard deviations of self-reported ratings across all 410 collected intervals and across both participants in the dyad were 6.95 ± 1.46 for valence and 6.14 ± 1.90 for arousal. This indicates that conversations were mildly pleasant and mildly energetic overall.
- Across all 84 participants, the difference between a participant's highest-related and lowest-rated interval was 3.07 ± 1.17 for valence and 3.54 ± 1.47 for arousal. This indicates that within a 20-min conversation, a participant's impression of the conversation valence and arousal tended to vary by slightly over 3 points on a 9-point scale.
- Across all 410 intervals, the absolute differences between the two individual participants' ratings for the same interval across all 410 intervals were 1.23 ± 1.22 for valence and 1.77 ± 1.57 for arousal. This indicates that the two participants tended to have similar but not identical impressions of the same conversation interval. However, we note that agreement between participants is not a requirement for the study design, and our secret prompts would occasionally induce different impressions since only one participant was aware of them.
- All possible valence and arousal values between 1 and 9 were present in the dataset, and there were multiple intervals where both participants in a dyad rated the same interval 9 for valence or 9 for arousal; however, there were no cases where both participants in a dyad rated the same interval as either 1 valence or 1 arousal. This indicates that while there were several intervals where the conversation was perceived very positively or energetically by both participants, there was no interval that was considered extremely negative or extremely low-energy by both participants.

Similarly, means ± standard deviations of observer-reported ratings across all 350 analyzed intervals and across both participants in the dyad were 5.86 ± 1.21 for valence and 5.72 ± 1.29 for arousal, indicating that observers also found the conversations mildly pleasant and mildly energetic overall.

### 3.1 Primary analyses

#### 3.1.1 Regression of self-reported valence and arousal

**Figure 4:**
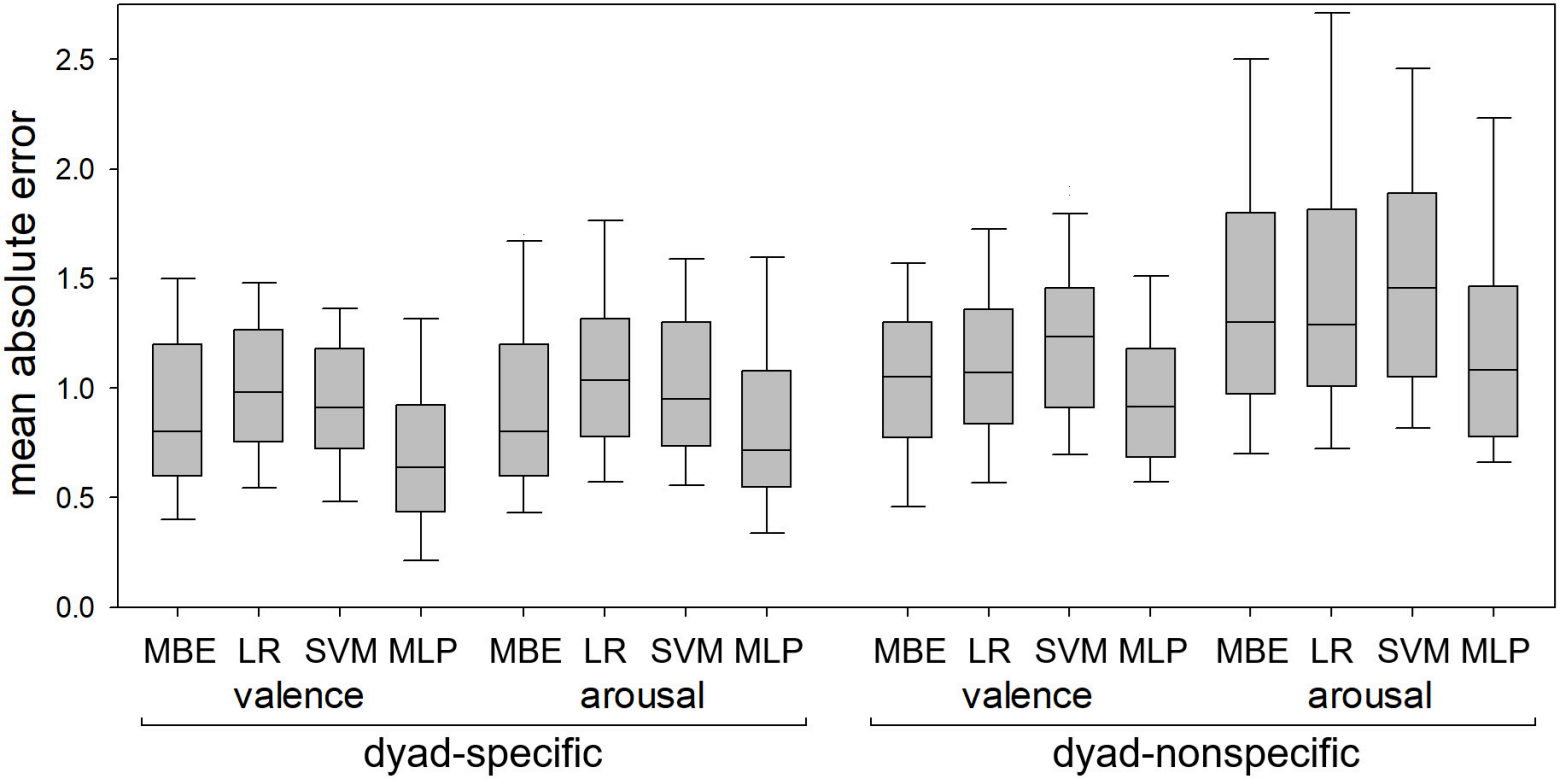
Mean absolute errors in dyad-specific and dyad-nonspecific regression of self-reported valence and arousal using the median-based estimator (MBE), linear regression (LR), support vector machine (SVM), and multilinear perceptron (MLP). The MLP consistently resulted in lower errors than LR and SVM but did not always outperform MBE.

**Figure 5:**
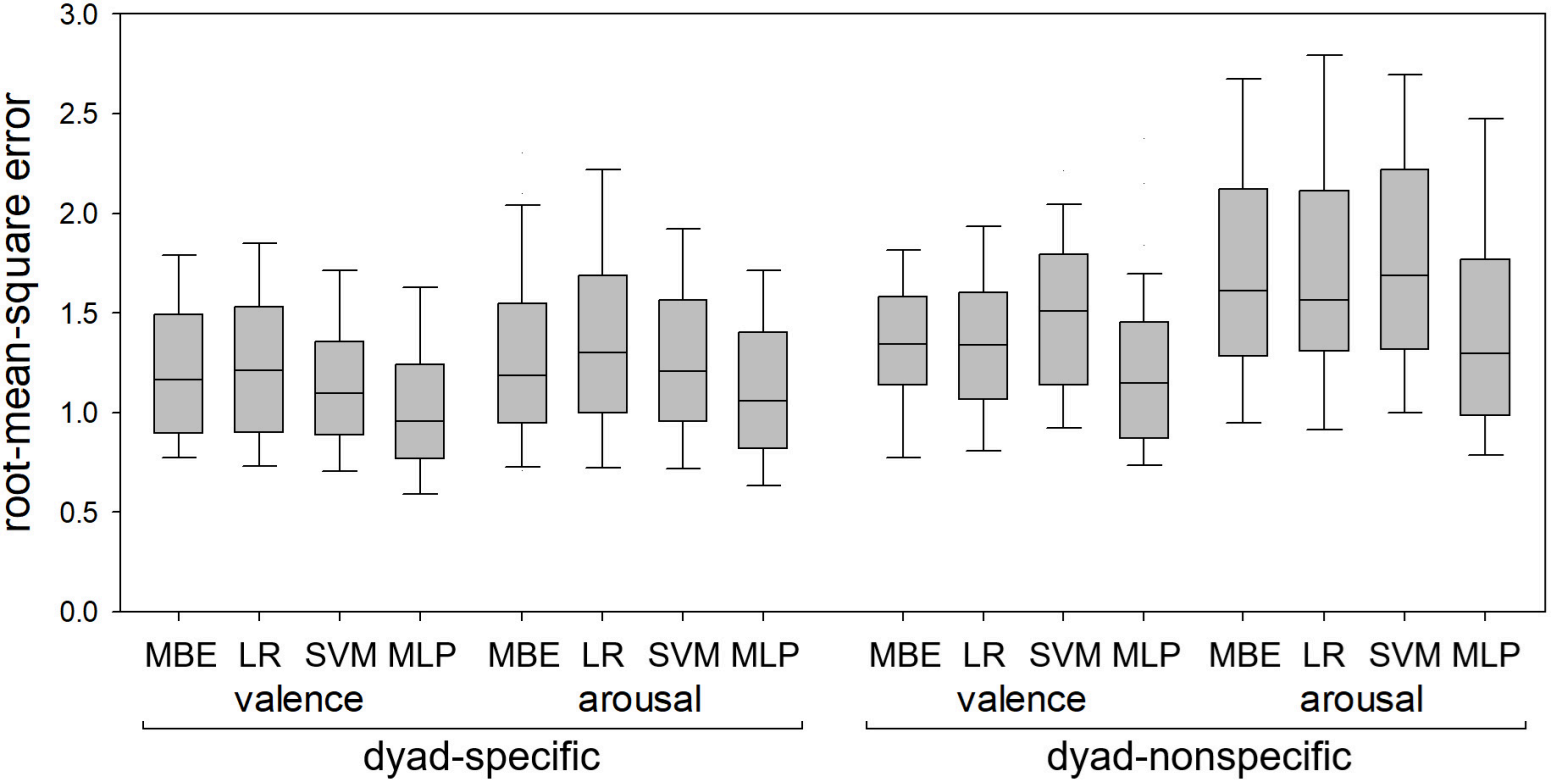
Root-mean-square errors in dyad-specific and dyad-nonspecific regression of self-reported valence and arousal using the median-based estimator (MBE), linear regression (LR), support vector machine (SVM), and multilinear perceptron (MLP). The MLP consistently resulted in lower errors than the other three methods.

Figure 4 shows MA errors in dyad-specific and dyad-nonspecific regression of self-reported valence and arousal for the four regression methods (median-based estimator, linear regression, SVM and MLP). Figure 5 shows RMS errors for the same problem. In each boxplot, the middle bar represents the median, bottom and top box edges represent 25th and 75th percentiles, and whiskers extend to the most extreme observation within 1.5 times interquartile range from the nearest quartile. All data are from 41 dyads.

All 8 RMANOVA found differences between regression methods. MLP-based regression resulted in lower RMS errors than the other three methods for both dyad-specific and dyad-nonspecific regression and for both valence and arousal (Figure 5), with *p* < 0.05 for all post hoc tests. For MA errors (Figure 4), however, MLP-based regression resulted in lower errors than linear regression and SVM in all cases (*p* < 0.05 for all post hoc tests) but only outperformed the median-based estimator for dyad-specific valence and dyad-nonspecific arousal estimation (not dyad-specific arousal estimation or dyad-nonspecific valence estimation).

In dyad-specific regression of self-reported valence and arousal using MLP, MRMR always resulted in lower errors than ranking with Laplacian scores. Conversely, in dyad-nonspecific regression of self-reported valence and arousal using MLP, ranking with Laplacian scores resulted in lower errors than MRMR. In dyad-specific regression, the lowest errors were achieved with 5–6 selected features; in dyad-nonspecific regression, the lowest errors were achieved with 10 features for MLP and 6–10 features for linear regression or SVM.

Finally, while the primary outcome metrics were MA and RMS errors calculated from the test datasets (where the finalized algorithms are applied to unseen data, as would be expected in practical use), comparing the MA and RMS errors between the training and test datasets allows us to estimate the degree to which overfitting may have occurred in the process. Table 1 thus shows how MA and RMS errors differ between training and test datasets for the three machine-learning-based methods.

**Table 1:** Comparison of errors on the training dataset vs. the test dataset for estimation of valence and arousal using the three machine-learning-based regression methods.

**Error type**	**Outcome**	**Method**	**Self-rated, dyad-specific**		**Self-rated, dyad-nonspecific**		**Observer-rated, dyad-nonspecific**	
			**Training**	**Test**	**Training**	**Test**	**Training**	**Test**
Mean absolute	Valence	MLP	0.59	0.72	0.89	0.98	0.72	0.72
		LR	0.83	1.01	1.09	1.11	0.95	1.00
		SVM	0.27	0.94	0.93	1.22	0.48	1.03
	Arousal	MLP	0.65	0.86	0.89	1.25	0.78	0.78
		LR	0.87	1.10	1.52	1.54	1.04	1.06
		SVM	0.61	1.01	0.58	1.60	0.42	1.08
Root mean square	Valence	MLP	0.93	1.09	1.14	1.19	0.92	0.95
		LR	1.07	1.26	1.41	1.38	1.19	1.19
		SVM	0.45	1.16	1.13	1.49	0.74	1.24
	Arousal	MLP	1.08	1.36	1.19	1.48	0.97	1.01
		LR	1.10	1.43	1.77	1.91	1.29	1.24
		SVM	0.85	1.28	0.95	1.86	0.67	1.29

MLP, multilinear perceptron; LR, linear regression; SVM, support vector machine.

Numbers presented are means over all training datasets or all test datasets in crossvalidation.

#### 3.1.2 Regression of observer-reported valence and arousal

**Figure 6:**
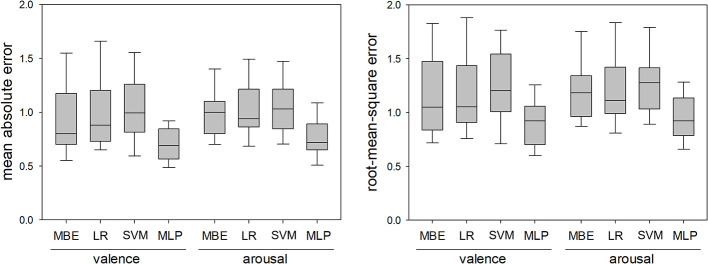
Mean absolute errors and root-mean-square errors in dyad-nonspecific regression of observer-reported valence and arousal using the median-based estimator (MBE), linear regression (LR), support vector machine (SVM), and multilinear perceptron (MLP). The MLP consistently resulted in lower errors than the other three methods.

Figure 6 shows MA (left) and RMS (right) errors in dyad-nonspecific regression of observer-reported valence and arousal for the four regression methods. All data are for 35 dyads. All 4 RMANOVA found differences between regression methods, and *post-hoc* tests found that MLP-based regression consistently achieved lower MA and RMS errors than the other three regression methods (*p* < 0.05 for all post hoc tests). As with self-reported valence and arousal, ranking with Laplacian scores consistently resulted in lower errors with MLP-based regression than MRMR, and the lowest errors were achieved with 10 features.

### 3.2 Secondary analyses

In the first analysis, physiological synchrony features were removed from the input dataset for MLP-based regression. Results are shown in Table 2 for RMS errors; results for MA errors were similar. As seen in the table, errors were significantly higher when synchrony features were removed from dyad-nonspecific estimation, but removal of synchrony features did not affect errors in dyad-specific estimation.

**Table 2:** Root-mean-square errors in the secondary analysis where valence and arousal were estimated using the multilinear perceptron with and without physiological synchrony features.

**Estimated variable**	**Root-mean-square error**		
	**No sync**	**With sync**	***p***
Self-reported valence, dyad-specific	1.04 ± 0.55	1.03 ± 0.40	0.81
Self-reported arousal, dyad-specific	1.22 ± 0.70	1.15 ± 0.51	0.84
Self-reported valence, dyad-nonspecific	1.33 ± 0.45	1.17 ± 0.47	< 0.001
Self-reported arousal, dyad-nonspecific	1.98 ± 0.84	1.46 ± 0.84	< 0.001
Observer-reported valence, dyad-nonspecific	1.00 ± 0.33	0.93 ± 0.28	0.13
Observer-reported arousal, dyad-nonspecific	0.99 ± 0.31	0.87 ± 0.26	0.013

Results shown as the mean ± standard deviation of individual participants' errors (for self-reported valence/arousal, *N* = 82) or individual dyads' errors (for observer-reported valence/arousal, *N* = 35). The *p-*value is the result of a paired *t-*test or Wilcoxon signed-rank test comparing error with and without synchrony.

In the second analysis, participant characteristics were added to the input datasets for dyad-nonspecific estimation of self-reported and observer-reported valence and arousal. This resulted in no significant changes to the results, and these results are not reported in detail.

Finally, Table 3 shows the five most important features for self-reported and observer-reported dyad-nonspecific regression of valence and arousal, obtained using ranking with Laplacian scores on the full dataset of 41 dyads (for self-reported values) or 35 dyads (for observer-reported values).

**Table 3:** Most important features for dyad-nonspecific regression of self-reported and observer-reported valence and arousal.

**Feature rank**	**Self-reported**		**Observer-reported**	
	**Valence**	**Arousal**	**Valence**	**Arousal**
1	EDA derivative DTW	EDA DTW	EDA Hausdorff distance	Heart rate Hausdorff distance
2	Skin temperature cross-correlation	Respiration DTW	EDA cosine distance	Mean temperature
3	Heart rate coherence	Respiration derivative DTW	Respiration cross-correlation	EDA complexity-invariant DTW
4	Mean heart rate	Respiration nonlinear interdep.	Respiration Hausdorff distance	Temperature nonlinear interdep.
5	Respiration complexity-invariant DTW	EDA complexity-invariant DTW	Standard deviation of respiration rate	Mean EDA

DTW, dynamic time warping distance; EDA, electrodermal activity.

## 4 Discussion

### 4.1 Primary analyses

#### 4.1.1 Comparison between regression methods

The MLP estimator was able to estimate valence and arousal with RMS errors lower than those of the median-based estimator in both dyad-specific and dyad-nonspecific regression of self-reported valence/arousal (Figures 4, 5) as well as in dyad-nonspecific regression of observer-reported valence/arousal (Figure 6). While the MLP estimator was also able to outperform the median-based estimator with regard to MA errors in several cases (Figures 4, 6), this was not always the case (Figure 4). However, we did a priori expect to find differences between MA and RMS error significance levels: the RMS metric has higher sensitivity to large errors since a squaring of errors is involved. When RMS but not MA errors are reduced, this implies that the regression algorithm is less likely to make large mistakes than the median-based estimator, but that the number of small errors is not reduced as much.

The MLP estimator also consistently outperformed linear regression and SVMs, which failed to outperform the simple median-based estimator. While this relatively poor performance of linear regression and SVMs may appear surprising, similar issues were observed in our prior work where (especially in dyad-specific estimation) many methods failed to outperform a median-based estimator (Chatterjee et al., 2021), so that finding is consistent with prior work. Comparison of training vs. test performance in Table 1 suggests that all three machine-learning-based methods experienced some overfitting (noticeably worse performance on test data than training data), especially in dyad-specific regression, but that the SVM appeared most prone to overfitting despite the use of feature selection methods. Thus, the poor performance of SVM is likely due to overfitting. Conversely, linear regression achieved worse performance than the MLP on both training and test data, and we believe that its relatively poor performance is because the relationship between physiology and psychology is highly nonlinear—as already known from single-user affective computing and group-level dyadic studies (see Introduction). Overall, any regression method for dyadic physiology clearly needs to be both nonlinear and robust with regard to overfitting.

#### 4.1.2 Comparison to prior work

For dyad-specific regression of self-reported responses, the difference between the MLP-based estimator and the baseline estimator in the current study is noticeably larger than the difference in our previous pilot regression study (Chatterjee et al., 2021). In that study, only a single conversation aspect (self-reported engagement) was estimated on a 1–100 scale; in dyad-specific estimation, the best regression algorithm yielded about a 5% improvement in MA error over the baseline median-based estimator and no improvement in RMS error. Conversely, in the current study, the dyad-specific median-based estimator achieved, for example, a mean MA error of 0.92 and mean RMS error of 1.22 for self-reported valence while the dyad-specific MLP-based algorithm achieved a mean MA error of 0.72 and mean RMS error of 1.03 for the same problem (i.e., about a 15% improvement). It is, however, unclear why the current dyad-specific results are better, as multiple aspects of the study protocol were improved from the previous study.

For dyad-nonspecific regression of self-reported responses, the difference between the MLP-based estimator and baseline estimator in the current study is also larger than the difference observed in our previous pilot regression study (Chatterjee et al., 2021). For example, in that study, median RMS errors for dyad-nonspecific regression of self-reported engagement were 14.0 with the median-based estimator and 12.6 with the best regression algorithm (i.e., about a 10% improvement). In the current study, mean RMS errors for dyad-nonspecific regression of self-reported variables are: for valence, 1.34 using the median-based estimator and 1.19 using the MLP-based estimator (i.e., about a 12% improvement); for arousal, 1.76 using the median-based estimator and 1.49 using the MLP-based estimator (i.e., about a 15% improvement). The same comparison cannot be made for regression of observer-reported valence/arousal, as there were no observer ratings in our previous study.

Beyond comparing numerical results between our current study and our previous one, we also emphasize that our current study used a more robust performance evaluation procedure. In our previous study (Chatterjee et al., 2021), we performed a training-test split for every tested regression algorithm and then reported results with the algorithm that achieved the lowest errors in the test group. However, this is not fully realistic, as manually selecting and reporting only the algorithm with the best performance in the test group introduces positive researcher bias and leads to unrealistically strong performance—in reality, we would not be able to preselect an algorithm for optimal performance on unseen test data. In this way, our current training-validation-test split is more realistic (as the best algorithm is selected before applying it to the test group) and the results are more applicable to real-world settings.

Aside from our previous study, we are aware of only one other dyadic regression study, which used electroencephalography to estimate self-reported valence and arousal ratings on 9-point scales during video watching (Ding et al., 2021). That study achieved mean MA errors of 0.78 for valence and 1.01 for arousal, which is similar to our dyad-specific MA error for valence (0.72) and worse than our dyad-specific MA error for arousal (0.87). However, that study's analysis procedure was very different from the current study, and results cannot be considered directly comparable.

Overall, our results indicate that conversation valence and arousal can be estimated on continuous scales from dyadic physiological responses and that estimation using MLPs is significantly more accurate than a simple median-based estimator when either self-report ratings or observer ratings are used as the ground truth. Additionally, the results are more promising than those of our previous study (Chatterjee et al., 2021) due to better performance in a more realistic evaluation procedure. Thus, from a fundamental scientific perspective, the results are encouraging.

### 4.2 Secondary analyses

First, adding participant characteristics (age, gender, personality traits) did not significantly reduce regression errors. A qualitative examination of MA and RMS errors obtained in MLP-based regression found that errors were actually slightly higher when these characteristics were included, and there is thus no compelling evidence to include them. Participant characteristics also did not improve accuracy in our previous dyadic classification work (Chatterjee et al., 2023), but were beneficial in our prior dyadic regression work (Chatterjee et al., 2021), so more research is needed to find a definitive result. It is possible, for instance, that they did contain some information but not enough to be selected by feature selection algorithms that had to select among 50+ features. Participant characteristics may be more helpful if they are, for example, not included as additional regression input features but instead used to select among different regression algorithms (e.g., use regressor 1 if participant has high neuroticism, but otherwise use regressor 2), though we currently have no evidence to support this suggestion.

Second, the most important features for regression (Table 3) differed for different variables and were extracted from all four physiological signals. While the #1 feature in Table 3 was always an EDA or heart rate feature, respiration and skin temperature commonly appeared as the #2 and/or #3 feature, suggesting that they may provide complementary information to the top-ranked feature. This mirrors the state of the art in single-user affective computing, where heart rate and EDA are the most commonly used autonomic nervous system responses while other signals like respiration are usually combined with heart rate and/or EDA rather than used in isolation (Aranha et al., 2021; Novak et al., 2012). Thus, all sensors appear important to the regression process and none can be obviously omitted to reduce hardware cost and setup time. This contributes to future research in this area by providing a justification for inclusion of the entire autonomic nervous system response sensor set in future studies. It also complements other studies in the field that suggest that, e.g., combined measures of sympathetic and parasympathetic physiological synchrony are more informative than measures of parasympathetic synchrony alone (Danyluck and Page-Gould, 2019).

Finally, most RMS errors increased significantly when synchrony features were removed from dyad-nonspecific regression (Table 2), and several synchrony features appeared among top 5 most important features (Table 3). Similar results were consistently observed in our prior work (Chatterjee et al., 2021, 2023). Thus, we believe that the additional signal processing and synchronization work needed to calculate synchrony features is justified by the contribution of these features to dyad-nonspecific regression, and that such features are likely to become standard in dyadic affective computing research. We believe that the lack of impact of synchrony features on dyad-specific regression (Table 2) is likely because there are much fewer available training data points in dyad-specific regression, making it harder for the regression algorithm to identify optimal coefficients without overfitting. With regard to specific synchrony features, the top-ranked features in Table 3 were DTW, derivative DTW, and Hausdorff distance; variants of DTW and Hausdorff distance also appeared multiple at other ranks in Table 3. Conversely, coherence and cosine distance each only appeared once in Table 3. Thus, while Hausdorff distance, derivative DTW and complexity-invariant DTW have (to our knowledge) not previously been used in dyadic affective computing research, they may be worthy of investigation in future studies.

### 4.3 Practical usefulness

As alluded to in our previous work (Chatterjee et al., 2021, 2023), a potential extension of the current work would be real-time automated conversation feedback: output valence/arousal values could be visually shown to participants. For example, a face on a screen could morph from frowning to smiling as valence increases. Some prototypes of such visualizations have already been presented, though most display raw physiological data (e.g., heart rate) rather than inferred conversation states (Feijt et al., 2021). Such feedback may allow participants to modify their behavior (e.g., by letting them know that the other person is not paying attention) and reach a more positive conversation outcome. This would be useful in areas like education (Dikker et al., 2017; Sun et al., 2020; Zheng et al., 2020) and mental health counseling (Bar-Kalifa et al., 2019; Kleinbub et al., 2019; Tschacher and Meier, 2020), especially for less experienced users (e.g., new teachers) who are less able to infer this information from subtle cues. A similar paradigm could be used in, e.g., cooperation to alert participants that they are not effectively working together (Balconi and Vanutelli, 2017; Hu et al., 2018), or in competition to dynamically adapt competitive games to suit both players (Darzi and Novak, 2021). Aside from real-time feedback, the same algorithms could be used to rate conversation or cooperation after it has happened—for example, to rate the effectiveness of educators or to identify people who work well together.

However, based on the current results, it is unclear whether automated estimation of valence and arousal would be practically useful. Two of the three methods presented (linear regression and SVMs) failed to outperform a simple median-based estimator. This is especially discouraging in dyad-nonspecific estimation where the state of a conversation is estimated based on states of other people's conversations—in that case, failure to outperform a median-based estimator means that the algorithm cannot do better than simply assuming the conversation is proceeding the same way as an average conversation. MLP did consistently outperform the median-based estimator: mean RMS values obtained using MLP were 10–20% lower than those obtained with the median-based estimator. The biggest improvements relative to the median-based estimator were obtained for estimation of observer ratings of valence (mean 21% lower with MLP) and arousal (mean 22% lower with MLP), as seen in Figure 6. This is in our opinion sensible, as the use of two trained researchers likely meant that the observer ratings were more consistent between dyads and easier to estimate with machine learning than self-reported values obtained from 41 dyads who may have interpreted the SAM in different ways. At the same time, it is debatable whether a 10–20% improvement in performance compared to a simple median-based estimator justifies the inclusion of multiple physiological sensors.

The discussion of whether a 10–20% improvement in performance is practically meaningful is complicated further by the lack of knowledge of what an “acceptable” regression error would be. While guidelines for acceptable classification errors do exist in affective computing (Hossain et al., 2025; McCrea et al., 2017), similar guidelines do not exist for regression, and the acceptable accuracies in classification are dependent on factors such as the consequences of classification errors (Hossain et al., 2025). A quick guideline from our previous work suggests that classification accuracy needs to be improved by approximately 10% to be reliably perceived by users in noncritical settings (McCrea et al., 2017); though classification is different from regression and the previous guideline is highly context-dependent, we do believe the 10–20% difference between MLP-based regression and the median-based estimator would likely be perceived by users.

At the same time, we do not believe that the MLP-based estimator, in its current state, is ready for practical deployment. While regression has the potential to provide granular information about users' psychological states, it has historically been less popular than classification in single-user affective computing (Novak et al., 2012), and the current level of MA and RMS errors does not guarantee that regression-based feedback would be more useful than, e.g., classification into 3 classes (e.g., low/medium/high valence). More broadly, the use of multiple physiological sensors decreases user comfort and relies on precise sensor positioning (Aranha et al., 2021), so errors may increase in a real-life scenario where, e.g., one sensor may shift position due to user movement and introduce artifacts. While practical usefulness cannot be fully evaluated without conducting an actual experiment where regression is used to provide feedback to users and its effects are evaluated, we thus currently recommend trying to further improve the accuracy of such regression before practically implementing such systems. For example, future studies may improve accuracy by incorporating other sensors such as speech emotion analysis and facial expression analysis, leveraging the relative benefits and drawbacks of physiological data.

### 4.4 Recommendations for future protocols

As mentioned, very few studies have applied regression algorithms to dyadic psychophysiological data. Our own previous study (Chatterjee et al., 2021) had several limitations that informed the current protocol, and we believe that further protocol improvements can be made using lessons learned from the current study.

First, we originally planned to include an equal number of acquainted and unacquainted dyads since the degree of familiarity between participants is known to influence physiological synchrony (Bizzego et al., 2020; Danyluck and Page-Gould, 2019). However, this was difficult for multiple reasons. Acquainted pairs were easier to recruit, as participants self-selected their partner, coordinated with each other to find a good session time, and reminded each other to attend the session. Conversely, solo volunteers had to coordinate with other solo volunteers either via a signup sheet or direct e-mail, and often could not find a time slot that worked for another solo volunteer. Without knowing the other person, they were also less motivated to attend, and several sessions were canceled because only one of the two participants attended. At the session itself, unacquainted dyads frequently had more difficulty talking continuously for 20 min as they were not sure what to discuss. As a result of these factors, we will either adjust our recruitment plan or focus on only acquainted pairs in future studies.

Second, we used secret prompts in this study because our previous study (Chatterjee et al., 2021) found that conversation engagement did not change much within a session when participants had completely freeflowing conversations. The secret prompts were meant to introduce more intervals with negative valence and “realistic” reactions since the other participant was not aware of them. However, many dyads were reluctant to follow the secret prompts; while this was not quantitatively evaluated, our observations suggest that most participants did try to follow the prompts but were sometimes not fully successful. We will thus consider alternatives such as acted disagreement in the future. One of our previous studies (Chatterjee et al., 2023) already used a simple form of acted scenarios (one very positive, one very negative), and we believe that more scenarios could be introduced to cover a wider range of valence/arousal/balance values.

Third, our analysis does not explicitly incorporate the fact that the two participants in a dyad may have different impressions of the conversation. When estimating self-reported valence/arousal, we estimated each individual participant's ratings, but physiological responses may look different when the other participant in the dyad agrees with the participant's view than when the other participant has a completely different impression. When estimating observer-reported valence/arousal, there was only one rating for each conversation interval, but the two participants may again have different impressions of the overall conversation, which may influence physiological responses. Situations where participants have different impressions are likely to be present in several—for example, a teacher thinking that they are engaging their students while the students are bored. We do believe that such situations are not common in our specific dataset (based on the characterization of conversation ratings in the Results), but future studies may specifically focus on such situations: for example, they could elicit them by asking participants to feign interest in a topic that they actually perceive as boring.

Finally, future analyses could incorporate additional outcome variables. While MA and RMS errors were used in previous dyadic regression work (Chatterjee et al., 2021; Ding et al., 2021), it would be possible to add outcomes such as R-squared, which was previously also used in single-user affective computing (Fairclough and Venables, 2006) and may provide an alternative basis for comparison.

## 5 Conclusion

Applying the MLP-based regression algorithm to physiological responses allowed us to estimate both self-reported and observer-reported valence and arousal of dyadic conversations with lower RMS errors than a relatively simple median-based estimator. This was the case in both dyad-specific regression (where the algorithm was trained using data from the same dyad) and dyad-nonspecific regression (where it was trained using data from other dyads). In most cases, MA errors were also lower using the MLP-based algorithm than with the median-based estimator. Furthermore, the difference between the MLP-based algorithm and the median-based estimator was larger than in our previous work, and MA and RMS errors were similar to those observed in other affective computing studies in other settings. Thus, from a scientific perspective, physiological measurements can be combined with regression algorithms to characterize dyadic conversations on the level of individual dyads and conversation intervals. However, as two other regression algorithms (linear regression and SVM) failed to outperform the median-based estimator, it should be emphasized that careful selection and design of algorithms is necessary to obtain good regression performance. In the long term, such regression approaches could be used as an alternative to the more popular classification approaches to obtain more granular information about the state of the conversation. This could be used in applications such as education and mental health counseling to both provide real-time feedback to interacting individuals (either face to face or online) and to rate the quality of the interaction after it has concluded. However, we believe that further work is necessary before such regression approaches can be deployed in applied settings, as the current performance level (in our opinion) does not yet justify the use of multiple physiological sensors in practice.

## Data Availability

Copies of questionnaires used in the study as well as all participants' demographics, extracted physiological features, and questionnaire answers are available on Zenodo: https://doi.org/10.5281/zenodo.10207814.
